# Supplementary material for: Compounding impacts of COVID-19, cyclone and price crash on vanilla farmers’ food security and natural resource use
Source: PLoS One. 2024 Oct 3;19(10):e0311249. doi: 10.1371/journal.pone.0311249 (PMC11449340; doi:10.1371/journal.pone.0311249)
Supplement: S1 File — (DOCX) [file pone.0311249.s001.docx]

# **S1 File: Semi-structured interview guide**

Name :

1. Gender :
2. Age :
3. Marital Status :

- Single
- Married / Living together
- Widowed
- Divorced

1. Are you the head of the household?

- Yes
- If not :
  - - Spouse
    - Daughter
    - Son
    - Other

1. How long have you been living here?
2. What is the highest level of school you have completed?
3. What is the size of your current household? Including only those who live 6 months or more in the household
4. What is the primary source of income in your household?

- Agriculture – Farming [to specify]
- Animal husbandry
- Teaching
- Tourist guides
- Government job
- Private job
- Wild product harvester (or hunter)
- Timber/wood working
- Other (specify):

1. Second sources of income :

- Agriculture – Farming [to specify]
- Animal husbandry
- Teaching
- Tourist guides
- Government job
- Private job
- Wild product harvester (or hunter)
- Timber/wood working
- Other (specify):

1. Of all the crises (cyclones, fires, droughts, covid-19, increase in fuel prices, vanilla price volatility and climate change) which happened the past 5 years (2017 – now) could you tell us which were the three most damaging to you and how serious they were?[Probes include the direct impact of the shock on their livelihoods, their current activities, food security, ...]
2. Which shocks have impacted your livelihood the most?
3. How did the shock (e.g. COVID-19) affect you? Can you tell us more about your experiences?
4. Can you tell us more about how you coped with the shock? What did you do to compensate the losses from the shock?
5. How did the shock affect the community’s forest use and forest access? [probes include: were there any changes in forest access and forest use, was their less enforcement of forest use restrictions in protected area and how did it affect them? How about forest clearing for cultivation (vanilla or rice farming)? How about timber harvesting or other forest uses that are legally prohibited inside the protected area? How did that impact the quality of local natural/forest habitats (e.g., biodiversity)?]. How about your household specifically?
6. Which of the recent natural disasters affected you the most in terms of food? [probe about effects on your eating habits and meal frequency?]
7. Can you tell us more about your challenges? How did you cope with these challenges? [probes include : To cope with the crisis, has the share of your income going for food changed? (Comparing the proportion of food expenditures prior to, and following the shock]
8. Besides natural disasters, which of the recent shocks affected you the most in terms of food? How did you cope?
9. Did one of the crisis/shocks particularly enhanced / encouraged human activities (e.g. switching to off-farm income such as small-scale mining etc.) or natural resource use in your community (i.e., increased forest product use, fishing, etc.)? Could you tell us more about it? How about your household specifically?
