# Supplementary material for: Compounding impacts of COVID-19, cyclone and price crash on vanilla farmers’ food security and natural resource use
Source: PLoS One. 2024 Oct 3;19(10):e0311249. doi: 10.1371/journal.pone.0311249 (PMC11449340; doi:10.1371/journal.pone.0311249)
Supplement: S2 File — (DOCX) [file pone.0311249.s002.docx]

# **S2 File: Scenario-based interview guide**

To better understand strategies that increase your resilience to shocks, we will discuss different possible futures based on scenarios we will provide. I want you to explain to me how you would react to each scenario. For example, what would be the likely impact on your livelihood and your household. We will suppose that nothing will change in the future, such as the price of goods, except for the current conditions I will present to you.

## Before starting with the scenario, it is better to talk a little bit about the current situation. Could you please tell us more about your current situation [about livelihoods, activities]? Do you face any difficulties [could you tell us more about it, what are your main concerns?] Could you tell us more about your strategies? How do you cope with them [regarding livelihoods, food security, ...]

## VANILLA PRICES

### *Increase*

I want you to imagine the next scenario: the price of vanilla has doubled. This implies that the vanilla trader will pay you twice as much as they do currently. Everything remains the same, except the fact that vanilla prices has doubled and last for five years. What will be the impact on you and your household? How will you react to it? Can you tell us more about your plans in the next few years (supposing that vanilla prices increase for the next five years)? How do you think that would affect your food security / livelihoods? How about the impact on your forest use / access?

### *Decrease*

Consider this fact: vanilla is at half price.

Now, imagine the next scenario: the price of vanilla has been reduced by half. Everything remains the same, but the price remains at the half price for the next five years. This implies that the vanilla trader will pay you half as much as they do currently. What will be the impact on you and your household? How will you react to it? Can you tell us more about your plans in the next few years (supposing that the vanilla prices decrease and continue for 5 years)? How do you think that would affect your food security / livelihoods? How about the impact on your forest use / access?

1. VANILLA DISEASE

Imagine that all vanilla plants here are affected by a disease, implying a large number of vanilla beans died, significantly reducing your harvest. Picture this vanilla disease lasting for five years. Can you elaborate on the impact this would have on you and your household? How would it affect your current activities? What measures would you take to cope with it? Furthermore, how might it influence your plans for the coming years? How do you think that would affect your food security / livelihoods? How about the impact on your forest use / access?

1. LAND DEGRADATION

Let’s imagine, that due to either human activities or a natural disaster the land has become degraded. For example, there is a sharp decline is soil fertility, soil is dry and damaged, crops cannot grow well, or there is erosion. Imagine that it continues for five years. How will this affect your future livelihoods? What will be the effect of this on your current activities? What will you do to cope with it? Could you tell us more about your future strategies? How do you think that would affect your food security / livelihoods? How about the impact on your forest use / access?

1. PANDEMIC RESTRICTIONS

Now, imagine that positive COVID rates go up and we are made to follow pandemic restrictions such as stay-at-home lockdowns, school and workplace closures, cancellation of events and public gathering, restrictions to transportation and so forth. Imagine that the situation lasts for 5 years. What will be the impact on you and your household? How will you react to it? Can you tell us more about your future plans and strategies? How do you think that would affect your food security / livelihoods? How about the impact on your forest use / access?

1. CLIMATE CHANGE

Imagine that the area you reside in is significantly affected by climate change, and this challenging situation is projected to persist for five years. Can you elaborate on the impact this would have on you and your household? How would it affect your current activities? What measures would you take to cope with the challenges posed by climate change? Furthermore, how might it influence your plans for the coming years, considering the changing climate patterns? How do you think that would affect your food security / livelihoods? How about the impact on your forest use / access?
