# Supplementary material for: Compounding impacts of COVID-19, cyclone and price crash on vanilla farmers’ food security and natural resource use
Source: PLoS One. 2024 Oct 3;19(10):e0311249. doi: 10.1371/journal.pone.0311249 (PMC11449340; doi:10.1371/journal.pone.0311249)
Supplement: S1 Table — (DOCX) [file pone.0311249.s003.docx]

# **S1 Table: Basic demographic information**

| **Category** | **Values** |
| --- | --- |
| **Gender** |  |
| Male | 36 (60%) |
| Female | 24 (40%) |
| **Age** |  |
| -35 | 14 (23%) |
| 35+ | 46 (77%) |
| **Education** |  |
| 0 years | 06 (10%) |
| Less than 5 years | 22 (37%) |
| Between 5 – 9 years | 27 (45%) |
| 10 – 12 years | 05 (8%) |
| **Primary source of income** |  |
| Farmers | 54 (90%) |
| Other | 06 (10%) |
