# Supplementary material for: Compounding impacts of COVID-19, cyclone and price crash on vanilla farmers’ food security and natural resource use
Source: PLoS One. 2024 Oct 3;19(10):e0311249. doi: 10.1371/journal.pone.0311249 (PMC11449340; doi:10.1371/journal.pone.0311249)
Supplement: S2 Table — (DOCX) [file pone.0311249.s004.docx]

# **S2 Table: Overview of the social impacts of the crises and local coping strategies and frequency of themes**

| **Crisis and Strategies** |  | **Description of the impacts of crisis and strategies** | **Exemplary statement from the interview** | **Frequency of themes reported** |
| --- | --- | --- | --- | --- |
| Crisis | Cyclone Enawo 2017 | The 2017 Enawo cyclone's impact caused significant devastation in the two villages, resulting in increased vulnerability due primarily to significant crop and home losses, disrupting livelihoods and food security. | “The cyclone devastated all my vanilla crops, resulting in a significant decline in income. Subsequently, we encountered food shortages as the cyclone also impacted all our crops meant for consumption.” | Crop losses: 68%  Homeless: 35%  Change in dietary habits: 67%  Households eating fewer meals per day than usual (2 instead of 3): 3% |
|  | COVID-19 pandemic | The restrictions imposed by the Malagasy government to contain the spread of COVID-19 had significant consequences on the country's farmers, both financially and in terms of food security. | “During the COVID-19 lockdown, we faced financial challenges due to the inability to work, coupled with the fear of violating regulations. The situation was tough, and on the food front, it was neglected.” | Income decrease: 54%  Total loss of income: 17%  Change in dietary habits: 46%  Farmers in a dire situation: 70%  Non-farmers witnessing a 50% decrease in their income: 7% |
|  | Vanilla price shock | The vanilla price shock affected both farmers and non-farmers by causing a decrease in their incomes and resulting in reduced employment opportunities within the village. Due to financial constraints, villagers were compelled to reduce their food consumption, with rice being the primary staple affected | “The decline in vanilla prices had significant repercussions on our livelihoods, as most villagers were facing financial problems as well. Consequently, we struggled to sell our goods, forcing us to lower prices. This compelled us to reduce our usual rice consumption.” | Income decrease: 87%  Food consumption reduction: 62%  Households facing a total loss of cash income: 10% |
| Strategies | Common | Crop diversification emerged as the primary strategy which consists in cultivating fast-growing annual crops, followed by daily wage labour and budget adjustment. In certain cases, household rescue to peer-to-peer lending while others sought loans from associations such as the Village Savings and Loan Association (VSLA). | “To address the situation, we had to resort to three-month crops.”    “The daily wage as a farm labourer was one of my strategies alongside farming.” | Crop diversification: 70%  Daily wage labor: 11%  Budget Management: 11%  Enhancing resilience by using agricultural practices responsive to local climate conditions: 45% |
|  | Distinct | In Andrapengy, the regulations on entry and usage of natural resources were more lenient the use of natural ressources appeared as a strategy to face the situation.  In Mandena, Marojejy National Park, has strict rules regarding natural resource usage and forest access. their reported strategies focused mainly on crop diversification (both crop sales and subsistence crops). | "Charcoal has become a second income-generating activity besides vanilla, and the situation has not improved, given the decline in vanilla prices... To this day, charcoal has become a first resort, and most people are doing it; some even rent cars to transport their charcoal from here to Antalaha...." |  |
