# Supplementary material for: Compounding impacts of COVID-19, cyclone and price crash on vanilla farmers’ food security and natural resource use
Source: PLoS One. 2024 Oct 3;19(10):e0311249. doi: 10.1371/journal.pone.0311249 (PMC11449340; doi:10.1371/journal.pone.0311249)
Supplement: Attachment — Submitted filename: Responses to reviewer comments.docx [file pone.0311249.s005.docx]

**Below we responded to the comments made by the two reviewers. Our responses are in blue, addressing all the comments point-by-point, and revised text in the resubmitted manuscript (in red, with line numbers in the manuscript without any tracked changes).**

*****************************************************

Reviewer #1: The paper studies the effects of individual and compounding crises (COVID-19, cyclone and vanilla price collapse) on smallholder vanilla farmers and local coping strategies in Madagascar. Applying semi-structured and scenario-based interviews across two case study villages with contrasting enforcement of forest regulations, authors found that the impact of the pandemic, combined with the cyclone event, disrupted livelihoods, resulting in income losses and food security challenges that exacerbated farmer vulnerabilities. The study draw attention to rural vulnerabilities in face of dual shocks of the covid19 and cyclone exposure.
Overall, the topic is important and the research question is valuable. However, I think the paper lacks systematic analysis to draw general conclusions about the impacts of dual shocks in local regions and the effectiveness of coping strategy through local forest exploitation in response to these shocks. In my opinion, the paper is more like a long report article in the media instead of a well-designed research article. Maybe the above is an issue of my personal understanding.
Response 1: Many thanks for your succinct summary of the paper and your positive feedback, as well as critiques.

Both reviewers point out that the qualitative nature of the study precludes generalizing to a broader population, a fact with which we agree. The study is intended to be qualitative in nature, obtained through open-answer, semi-structured interviews and scenario-based interviews, which allowed us to glean nuances of individual perceptions of risks, challenges, and adaptive strategies for coping with crises. Although we have included more quantitative evidence to support how often participants reported similar themes (throughout the results section and also in S2 Table), we emphasize that there are risks to over- and mis-interpreting the results if one focuses too narrowly on quantitative results, and these are not to be generalized to larger populations. We also emphasize the importance of even a single report, rather than focus only on majority perceptions. Throughout the manuscript, we made revisions to increase the reporting of empirical findings. There are additions to the methods providing more background on the field of context-based narrative and thematic analyses, and interpretations of our results into this broader field in the discussion. We also point out the limitations, specifically that we should not generalize to broader regions from our results, especially not based on the reported frequency data. Lastly, we edited the language such that we hope the reviewer and readers will not perceive the writing style as media rather than academic, though there should not be such a stark dichotomy.

To give some specifics:

First, we outlined our research aims in lines 68-81 and edited the wording to increase clarity:

This research aims to study the individual and compounding effects of global crises (COVID-19, cyclones and vanilla price collapse) on farmers' lives across two case study villages in northeast Madagascar. Using semi-structured and scenario-based interviews, we identified various mechanisms by which these cascading crises can consecutively and concurrently impact the livelihoods and food security of farmers in these case villages. We further explored the knock-on impacts on natural resource use and biodiversity. Finally, we report the various strategies employed by farmers to cope with these challenges. By focusing on the context-based specific realities of the case study villages, this article contributes to a better understanding of the challenges faced by farmers in this region of Madagascar and provides wider lessons for similar contexts in low-income countries. We explored these research aims using a qualitative approach. Qualitative approaches can help provide detailed, vivid, and context-sensitive descriptions of the data and and gain an in-depth understanding of the context-specific challenges and nuanced perspectives of place-based actors [14].

Second, to more clearly describe how we address these research aims, we carefully elaborated on the semi-structured interview guide and scenario-based guide (please see lines 96-113).

The semi-structured interview guides (S1 File) consisted of 18 questions that covered participant basic demographics, including age, household size, education level, and income sources. These interviews also examined the impacts of various crises, such as cyclones, COVID-19, and fluctuations in vanilla prices on livelihoods and food security. Additionally, participants were asked about the coping strategies they adopted in response to each crisis and the potential effects of each crisis and their compounding effects on the use of natural resources.

The scenario-based interview guide (S2 File) covered six hypothetical scenarios encompassing the status quo, an increase or decrease in vanilla prices, land degradation, diseases affecting vanilla cultivation, the restrictions during COVID-19, and extreme weather events such as cyclones. For each scenario, participants were prompted to share the anticipated effects of these crises on their lives, and their coping strategies. This method allowed us to delve into the nuanced decision-making processes of farmers, including a long-term perspective on adaptive responses they have used and their perspectives in the face of uncertainty. Respondents were prompted to speak on any of the crises they chose to discuss, without restricting the timeframe.

Third, we analyzed these data using both thematic and content analysis methods. Thematic analysis in qualitative research provides a rich, detailed, complex account of data. As Braun and Clarke [1] put it, thematic analysis is a method for “identifying, analyzing and reporting patterns (themes) within data. It minimally organizes and describes the data set in (rich) detail.” Thematic analysis involves identifying and understanding key themes in the data and how they relate to one another. Eponymously, the *themes* derived from the data actively construct the patterns of meaning to address our research aims. In short, themes are ‘a patterned response or meaning’ derived from coded data that represent overarching ideas embedded within the larger data set.

We have added more details in lines 127-150, and also suggested how our results should be interpreted:

We analyzed the data using thematic and content analysis methods [16,17]. Thematic analysis involved identifying and analyzing themes in the data, including initial coding of meaningful units, searching for common themes, revising and defining themes, and interpreting the results [16]. We specifically searched for any themes and patterns indicating the potential effects of various crises, including COVID-19, cyclones, and vanilla price fluctuations on household livelihoods and food insecurity, the effects of these crises on the use of natural resources and biodiversity, and the diverse strategies adopted by farmers. The aim is to uncover and understand key themes in the data and how they relate to one another. The content analysis involved a systematic analysis of the key themes identified from the thematic analysis [17]. It focuses on the reoccurrence of themes at a more surface-level of analysis i.e. their frequency.

Researchers and readers should use caution when interpreting such frequencies, however [18]. Frequencies may help get a better feel for how often themes occur within the specific sample, and advance qualitative research within a discipline that are not familiar with or oriented to qualitative research. Nevertheless, incorporating frequencies in qualitative research may misrepresent data as not every interviewee is asked the same questions in the same way [19]. Number can also detract from the more valuable, detailed, and nuanced insights uncovered from qualitative research [19]. Similarly, though a theme or response that only occurs once may seem unimportant based on frequency of occurrence, it may represent the unique challenge, solution, idea, or theory that could influence social, anthropological, and policy change. Lastly, readers should not interpret frequencies as generalizations to broader populations; instead, one should assess the transferability of the findings, sometimes also referred to as analytic generalization or case-to-case generalization [18,19].

Fourth, to explain the systematic nature of our analysis and the rigor with which the data were treated, we added a paragraph in lines 151-160 to emphasize our efforts to ensure the final analytic framework represents a credible account of the data.

Coding was conducted iteratively by HRH to generate codes and themes that summarized and captured the essence of the responses. a second coder (OSR) used the draft coding scheme to independently code five randomly selected sample transcripts. To ensure consistency in coding and mitigate the subjectivity of a single coder, we calculated the intercoder reliability, the Cohen’s Kappa coefficient which is a numerical measure of the agreement between different coders regarding how the same data should be coded [20]. We considered a coefficient with a value of 0.70 or higher acceptable. Codes that did not meet this threshold were jointly reviewed, and conflicting interpretations were resolved by either merging codes or refining code definitions. These analyses were conducted with NVivo 12 software.

The following are more detailed comments:

1. It will be helpful to do more general analysis on the data based on interviewers’ answer. In the current version, authors just simply list quote out the direct answers to the survey questions. It is not clear how representative one person’s answer is in the aspect of the whole village or the local regions. Or how the surveyed individuals’ answers differ from each other.

Response 2: We thank the reviewer for this suggestion, and have taken the following steps. To provide the readers with more quantitative evidence, a table is included in the supplementary materials (Table S2), synthesizing the frequency or occurrence of specific themes. We present these results as descriptive statistics and emphasize caution in reading into significance or representativeness as in quantitative methods. A theme that only appeared once doesn’t necessarily indicate that it is less important.

As described above, we provided more details on the thematic and content analyses we conducted to address our research questions. The purpose of these qualitative approaches is to provide in-depth explanations and meanings rather than generalizing findings. Our paper provides detailed, vivid, and context-sensitive descriptions of the data in our manuscript, which is essential in qualitative research. The verbatim quotes from the transcripts not only provide evidence of the general themes that respondents reported, but deep, rich, and context-specific evidence, both of which support our findings.

In the methods, we provide more background on the following papers to further develop the theory, and describe how frequencies can complement and enhance narratives and improve transparency (see lines 138-147). We especially build on the works of Black 1994; Sandelowski 2001; Neale et al 2014; Maxwell 2010; Hannah and Lautsch 2011 (references provided at the end). More specifically, frequencies may help get a better feel for how often themes occur, and advance qualitative research within a discipline that are not familiar with or oriented to qualitative research. Nevertheless, incorporating frequencies numbers in qualitative research may mislead readers or replace the actual description of evidence.

To be more specific in this response, and as summarized in the methods of the revised manuscript, Maxwell (2010, p. 479) articulated the potential pitfalls of overinterpreting frequency data derived from qualitative research:

Numbers can lead to the inference (by either the researcher or the audience) of greater generality for the conclusions than is justified, by slighting the specific context within which this conclusion is drawn. A particular setting or sample may be unrepresentative, and a facile reading of quantitative results may lead a reader to ignore this limitation. Qualitative research is intrinsically local, and any claims for the generality of its conclusions rely on a different kind of argument from that of quantitative research, what Yin (1994) called analytic rather than statistical generalization.

Generalizations can be made from qualitative research, but just not in the same way as quantitative results are (which aims to generalize findings to a larger population). The type of generalization that is applicable to our qualitative analysis is transferability which is sometimes also referred to as analytic generalization or case-to-case generalization. The question is “to what extent are these results transferable to other settings?’ For example, a development practitioner who is designing a livelihood intervention might want to know: “Are these patterns also true in the context I work with and what would be the most effective policy levers increasing local resilience?” When readers feel as though this can be the case – when they believe that research overlaps with their own situation and/or they can intuitively transfer the findings to their own action –, then the research can be said to generalize through transferability.

We emphasize this in our revised manuscript both in the methods and in the policy implications:

Lines 147-150

Lastly, readers should not interpret frequencies as generalizations to broader populations; instead, one should assess the transferability of the findings, sometimes also referred to as analytic generalization or case-to-case generalization.

Lines 482-485:

Given the relatively small sample and our qualitative approaches, we cannot provide generalizations to the population from which the sample was drawn. Our research findings can however be transferable to other settings and provide wider lessons for similar contexts in low-income countries.

Throughout the discussion, we shed light on the transferability of our results by reflecting on how our results compare with findings in similar contexts. For instance:

Lines 409-415:

The impacts of government-imposed lockdowns and restrictions on livelihoods were severe, affecting nearly 70% of households. Similar magnitudes of impact were observed in other parts of Madagascar [22] as well as among Ugandan households, where two-thirds reported income losses during the pandemic [23]. Farmers encountered challenges due to travel restrictions that hindered their ability to sell produce freely, resulting in limited market access and reduced income [23] .

Lines 449-456

Simultaneous shocks can magnify vulnerabilities, leading to cascading impacts on livelihoods and well-being. These findings are in line with previous studies reporting that the pandemic posed new challenges to livelihood strategies while complicating disaster management during cyclone season [29,30]. The intersection of the pandemic and cyclone Amphan in Bangladesh highlighted the need to consider complex temporalities of disasters [31]. These findings are also consistent with similar studies documenting the compounding impacts of multiple crises on vulnerable populations [32,33].

Lines 485-496

Our study warrants the need for targeted support systems that integrate disaster risk reduction, livelihood diversification, and market resilience strategies to foster long-term sustainability and strengthen the resilience of households facing multiple crises.

Given the vulnerability of households heavily dependent on single cash crops like vanilla, policymakers should promote diversification of income sources and agricultural practices as recommended in other similar contexts [34,35]. In addition, disaster preparedness and risk reduction efforts can be strengthened through policy interventions. Investing in early warning systems, promoting climate-resilient infrastructure, and fostering community-based disaster management plans can enhance local communities’ ability to cope with future cyclones and other environmental challenges [36,37].

2. Related to the first point, it will be helpful to describe the heterogeneous patterns in households’ consumption and income in response to the dual shocks. Are the magnitudes of their income drops the same or different? What factors affect their different responses?

Response 3: We appreciate this suggestion. In Table S2, we synthesized the frequency or occurrence of specific themes to show their heterogeneity and give broader indications of how prevalent the various themes were (e.g. percentage of participants that mentioned crop losses or income decrease from the various crises). We, however, caution readers that this does not equate to magnitudes of lost income. Our qualitative research design was chosen for this specific context, and with the current data we are unable to provide quantitative estimates on the magnitude of the impacts of various shocks or quantitative comparisons of these impacts. In this rural farming community, few households keep records of their revenues and expenses, but rather live day-to-day off their subsistence crops, what they can sell, and what they can earn from wage labor.

Therefore, rather than try to quantify the magnitude of changes in income and consumption, we asked scenario-based questions that helped elicit responses which capture the subjective experience of the respondent. This allowed us to gain insights outside of what we might have pre-supposed to be important, such as income. For example, a key finding was that when income was impacted by a crisis, respondents told us that community members turned to forest products, which is more important to understanding adaptive coping strategies than whether respondents ‘broke even’ or how much lost income they sustained.

3. How representative are the conclusions from these two villages on the general condition in Madagascar. What the potential application of the shock coping strategy in other regions?

Response 4: Thank you for this comment. We review our results in light of other literature on Madagascar and globally throughout the discussion (please see our response 2 above).

We emphasize in the discussion (lines 482-485) that the generalizations possible from our qualitative analysis should be framed as transferability, which is sometimes also referred to as analytic generalization or case-to-case generalization (please see our response 2 above).

Reviewer #2: Lack of Quantitative Data: The study relies solely on qualitative data from semi-structured and scenario-based interviews. While rich in detail, the absence of quantitative data limits the ability to generalize findings and assess the magnitude of impacts.

Response 5: Many thanks for your comments. These comments are largely in line with those of reviewer 1 above, and both of these reviewers provided helpful suggestions that encouraged us to include more quantitative evidence to the thematic analysis of transcript data (please our Response #1, 2 and 3 above). We also elaborate throughout the methods and discussion about how such descriptive statistics can help to understand the potential prevalence of themes, but that even infrequent themes can be incredibly important, and frequent themes may be misleading. We will not re-iterate the revisions made to the manuscript here, but believe we addressed them above.

Limited Geographic Scope: The research is confined to two villages in the SAVA region. This narrow geographic focus may not capture the broader variations and challenges faced by farmers in other parts of Madagascar or other low-income countries.

Sample Size: The sample size of 60 participants (30 per village) is relatively small. This may affect the representativeness of the findings and limit the robustness of the conclusions drawn.

Response 6: These are important points which we highlight in the methods and discussion. Specifically, we emphasize that our goal is not to generalize beyond our sample frame; rather, our objective is to provide deep contextual information on the true impacts of concurrent and compounding crises for these rural communities and how they cope. With that information, we can a) compare to other studies to determine if these communities showed similar patterns to other studies using different methods, and b) provide context-dependent, place-based policy recommendations which are transferable to similarly vulnerable populations.

Qualitative research necessitates having a small sample because of the detailed and intensive work required for the study. Purposive sampling is common in qualitative research. Particular individuals are chosen with characteristics relevant to the study who are thought will be most informative by local leaders. In our study, we aimed to generate a representative sample by creating community maps of each sector of each village, and sampling across sectors to ensure peripheral households were also included.

Please see our previous responses (response 2 and 4 above) on the generalizability or transferability of our findings to other contexts.
Temporal Scope: Interviews were conducted over a short period (January and February 2023). This limited timeframe may not fully capture the long-term impacts of the crises or seasonal variations in livelihoods and coping strategies.

Response 7: Thanks for the comment, it made us realize we did not explain that the scenario-based interview guide allowed us to capture as deep a timeline as the respondents felt was important. The interviews revealed a depth that could not be captured with most standardized quantitative surveys that have only a snapshot of time. Though we only conducted interviews during a short time, the guide asked respondents to think of times in the past when they suffered from crisis situations and how they coped. Respondents frequently told of the cyclone in 2017, the pandemic in 2020-2022, etc. We emphasized this in our results.

For instance, in lines 235-241

A compelling case illustrating this situation was reported in Andrapengy village, where a family recounted their experience:

*"There has been a considerable decrease in the amount of rice consumed. Previously, we used to consume 4 cups of rice around the same time of the year, but we had to reduce it to 2.5 cups once the COVID-19 pandemic struck... At times, we even reduce the frequency of meals, resulting in only the children having a meal in the evening while the adults go to bed on an empty stomach."* (Male, 41 years old, Andrapengy)

In lines 252-260

Among the surveyed households, 68% reported crop losses, particularly essential crops like vanilla and cloves, due to strong winds and heavy rainfall. Furthermore, the destruction of homes left 21 out of 60 households homeless, leading to social instability as they sought new places to live or relied on the support of nearby families. One resident shared the psychological distress caused by the cyclone, stating:

*"My house was ravaged, my vanilla crops were destroyed, and the bananas, which were also devastated, were not yet available or ripe. It almost drove me insane.”* (Male, 53 years old, Mandena)

In lines 337-340

In the aftermath of COVID19 and the cyclones, it was adopted by at least 50% of households. During the prolonged declines in vanilla prices, up to 70% of residents opted to diversify their crops for selling purposes.

Potential Bias in Participant Selection: Although participants were randomly selected, the reliance on local authorities and key informants for the sample frame may introduce selection bias, potentially influencing the diversity and representativeness of the sample.

Response 8: We acknowledge this potential limitation / source of bias in lines 482-485). As explained in the methods (lines 114-116), the key informants were essential in building the sampling frame (complete list of households in our study villages), as full census data are generally lacking or outdated in these rural contexts. Selection bias is therefore less of a concern as we randomly selected participants from the sampling frame.

Ethical Considerations: The study obtained verbal consent, which may be less robust than written consent in ensuring participants fully understand the research implications and their rights. This could raise ethical concerns regarding informed consent.

Response 9: Verbal consent was approved by our ethics review board (lines 120-126) because it was deemed that our data collection would not elicit such sensitive information that would warrant the need for written consent. While a written consent may seem better in other settings, in practice in this case, written (i.e., signed) consent raises concerns in participants, in our experience. With a long colonial history of exploitation, Malagasy people have a well-documented legacy of experiencing land-grabbing by foreigners as well as nationals. With low literacy rates in these and similar communities in Madagascar, not knowing what a document says and being asked to sign it is threatening and off putting; thus, for this context, it was not necessary. (47% of the participants have less than 5 years of schooling on average).

Generalization of Findings: The unique socio-economic and environmental context of the SAVA region, especially its heavy reliance on vanilla farming, may limit the applicability of the findings to other regions with different agricultural and economic profiles.

Response 10: In addition to our previous responses in which we caution the reader from drawing too general a conclusion from our results, we provide review of how similar patterns are observed in other cash cropping systems in Madagascar and globally (please the section on the policy implications, lines 482-504).

Absence of Longitudinal Data: The study does not incorporate longitudinal data to track changes over time, which would provide a more comprehensive understanding of the evolving impacts of crises on livelihoods and food security.

Response 11: Please see our previous response @7 above.

Analysis Limitations: While thematic and content analysis were employed, the article does not discuss potential limitations of these methods or how the researchers addressed issues of reliability and validity in the qualitative data analysis.

Response 12: As discussed above, we added one paragraph in the methods section to outline how we have assessed the reliability of our coding (lines 151-160).

Coding was conducted iteratively by HRH to generate codes and themes that summarized and captured the essence of the responses. a second coder (OSR) used the draft coding scheme to independently code five randomly selected sample transcripts. To ensure consistency in coding and mitigate the subjectivity of a single coder, we calculated the intercoder reliability, the Cohen’s Kappa coefficient which is a numerical measure of the agreement between different coders regarding how the same data should be coded [20]. We considered a coefficient with a value of 0.70 or higher acceptable. Codes that did not meet this threshold were jointly reviewed, and conflicting interpretations were resolved by either merging codes or refining code definitions. These analyses were conducted with NVivo 12 software.

We also added a paragraph discussing the generalizability, or more appropriately: transferability, of our findings in lines 147-150 and lines 482-485.

Lines 147-150

Lastly, readers should not interpret frequencies as generalizations to broader populations; instead, one should assess the transferability of the findings, sometimes also referred to as analytic generalization or case-to-case generalization.

Lines 482-485:

Given the relatively small sample and our qualitative approaches, we cannot provide generalizations to the population from which the sample was drawn. Our research findings can however be transferable to other settings and provide wider lessons for similar contexts in low-income countries.

External Validation: The study lacks a discussion on external validation of the findings. Incorporating perspectives from other regions or cross-referencing with existing quantitative studies could strengthen the credibility and relevance of the conclusions.

Response 13: Throughout the discussions, we compared our study findings with previous literature and similar contexts, as described above. We provided quantitative figures from our results, where appropriate, and point to other studies that have quantitative results as well.

Coping Strategies Analysis: The analysis of coping strategies might benefit from a deeper exploration of the effectiveness and sustainability of these strategies. The study does not sufficiently address whether the identified coping mechanisms are viable long-term solutions.

Response 14: We appreciate this point and add to the paragraph on limitations that a future study should investigate quantitatively the impacts of the crises, the trade-offs with coping strategies and their long-term viability (lines 504-506). However, it was beyond the scope of our study.

Future research avenues could include quantitative estimation of the social impacts of various crises, and investigation of the long-term viability of key policy interventions aimed at increasing local resilience.

Integration of Local Knowledge: Although the study incorporates local informants, it does not elaborate on how local knowledge and cultural practices were integrated into the analysis. A more explicit discussion of this integration could enhance the contextual relevance of the findings.

Addressing these comments in the revision could provide a more comprehensive and generalizable understanding of the impacts of compounding crises on farmer vulnerability and resilience in Madagascar and similar contexts.

Braun V, Clarke V. Using thematic analysis in psychology. Qualitative Research in Psychology. 2006;3(2):77-101. doi: 10.1191/1478088706qp063oa.

Response 15: We appreciate the value of local knowledge and practices, and this study was a preliminary look into some of those practices. An in-depth contextually-based study co-created with these knowledge keepers is a next step, but we cannot over interpret the results of our current data. We allude to this in the limitations/future studies section (lines 504-506) without overstating what our current/future projects can do.

In general, we appreciate the reviewers’ efforts, and we feel that by incorporating their feedback, we should be able to address their concerns and we believe the readers will have a better grasp of the value of this study.

References provided in the response letter (also included in manuscript where appropriate)

Black, N. (1994) Editorial: Why we need qualitative research, Journal of epidemiology and community health, 48, 5, pp. 425-426.

Hannah, D. R. and Lautsch, B. A. (2011) Counting in qualitative research: Why to conduct it, when to avoid it, and when to closet it, Journal of Management Inquiry, 20, 1, pp. 14-22.

Maxwell, J. A. (2010) Using Numbers in Qualitative Research, Qualitative Inquiry, 16, 6, pp. 475-482.

Neale, J., Miller, P. and West, R. (2014) Editorial: Reporting quantitative information in qualitative research: guidance for authors and reviewers, Addiction, 109, 2, pp. 175-176.

Sandelowski, M. (2001) Real qualitative researchers do not count: the use of numbers in qualitative research, Research in nursing and health, 24, 3, pp. 230-240.
